# Reading and comprehension: phoniatric assessment in students with reading difficulties

**DOI:** 10.1016/j.bjorl.2021.05.014

**Published:** 2021-07-21

**Authors:** Vanessa Magosso Franchi, Mônica Elisabeth Simons Guerra, Beatriz Cavalcanti Albuquerque Caiuby Novaes, Mariana Lopes Favero, Sulene Pirana

**Affiliations:** Pontifícia Universidade Católica de São Paulo (PUC-SP), São Paulo, SP, Brazil

**Keywords:** Academic developmental disorder, Child development, Dyslexia, Comprehension

## Abstract

**Introduction:**

Reading is a highly refined skill that encompasses two main components: decoding graphic symbols and understanding the written message. These aspects generally develop together, but reading comprehension is a much more complex process, sustained not only by the identification of written words and vocabulary but also by language systems, such as syntax and general knowledge. Although there is a well-established technique for performing the phoniatric assessment, there is no common use of tests that assess reading comprehension or the association of this information with other assessment data.

**Objective:**

The objective of this study is, in the context of the phoniatric consultation, to evaluate the reading and retelling in children with relevant reading difficulties and to correlate the decoding and comprehension problems with the alterations observed in auditory and visual perceptual tests, pointing out the evidence that best contributed to the differential diagnosis of these subjects.

**Methods:**

Starting from a population of 301 children enrolled in the 4th and 5th grades of elementary school, 13 children with evident reading and writing difficulties were evaluated regarding the reading and retelling tasks and separated into groups according to the problem of decoding, fluency, and comprehension. Reading performance was correlated with the performance in visual and auditory perceptual tests and based on the similarity analysis, the tests considered to be the most relevant in the diagnosis process of these children were identified.

**Result:**

The results suggest that the tasks: naming of figures, repetition of numbers in reverse order, figure copying, syllabic synthesis, phonemic synthesis, rhyme, and phonemic manipulation altogether contribute to diagnosis and multidisciplinary intervention aspects.

**Conclusion:**

Some tasks are more relevant to the diagnostic process of children with complaints of learning difficulties in reading.

## Highlights

• Learning difficulty.
• Phoniatric assessment in learning disorders.
• Phoniatrics and reading and writing learning disorders.
• Phoniatric assessment and neurodevelopmental disorder.

## Introduction

According to PISA (International Student Assessment Program) 2018, Brazil performed below the average of most OECD (Organization for Economic Cooperation and Development) countries, ranking 59th among the 70 assessed countries. According to UNESCO data published in 2017, 22.7% of children enrolled in the 5th year of Elementary School were below the expectations for reading skills in Brazil.^1^

Failures in learning how to read and write are caused by a heterogeneous group of problems that impact academic performance.^2^ Understanding the causes of the difficulties faced by these children and performing the differential diagnosis contributes to the improvement of this performance.^2^

The term ‘learning disorder’ is used for learning difficulties characterized by performance below the expectations for age, intellectual level and schooling in students who have favorable conditions and contexts for learning.^3^, ^4^ It affects 5%–15% of school-age children, in different languages ​​and cultures, and is considered a serious problem due to the great impact on family life, such as low self-esteem, socialization problems and school dropout, which significantly impact adult life.^4^, ^5^, ^6^

Reading is a highly refined skill that comprises the joint development of decoding graphic symbols and the comprehension of the written message; these two components usually develop together and, therefore, reading comprehension impairment can develop as a result of a deficit in any of these domains, involving cognitive and linguistic neural areas.^7^, ^8^, ^9^

Phoniatrics has been an area of ​​expertise in otorhinolaryngology since 2006 and works in the differential medical diagnosis of language and learning problems.^10^, ^11^ The phoniatric assessment is a comprehensive clinical study that involves the investigation of several factors in the individual's life, both environmental factors such as individual and family neurobiological tests and includes standardized or non-standardized tests.^10^, ^11^

The aim of this study was, in the context of the phoniatric consultation, to evaluate the reading and retelling in children with relevant reading difficulties, who could potentially seek a phoniatric specialist’s evaluation and group them according to their performance, aiming to correlate decoding problems, fluency and understanding with the alterations presented in auditory and visual perceptual tests, suggesting the tests that best contributed to the differential diagnosis of these children.

## Methods

### Ethical considerations

The present study was based on a Master's degree thesis and was approved by the Research Ethics Committee, according to Resolution N. 510/2016 of the National Health Council, under Opinion n. 2.572.678.

### Participants

This cross-sectional descriptive study identified 13 children with significant reading difficulties. The study was based on a population of 301 children enrolled in the 4th and 5th years of elementary school in one of the best public schools in the ranking of the state of São Paulo, Brazil, with a Basic Education Development Index — IDEB^12^ of 8.1 in 2017. Of these, 166 were included in the study and met the criteria: age between 8 years and 12 years and 11 months, normal otorhinolaryngological and audiological evaluation, normal visual acuity, consent form signed by parents or guardians and assent form signed by the child and absence of previously diagnosed diseases, such as genetic syndromes, intellectual disability, autism spectrum disorder and attention-deficit/hyperactivity disorder. Subsequently, the teachers of the 166 children answered a questionnaire (Table 1) to identify those who had great difficulty in reading to the point of compromising their school performance, despite the teacher’s dedication, so that a professional evaluation by a specialist in phoniatrics could be indicated.

**Table 1:** Questionnaire for the teachers.

Which children in your classroom have a lot of difficulty in learning how to read, such as: lack of fluency or difficulty understanding the text?
Which children are not literate yet?
Which children show impacts on their school performance due to the difficulty? (Grades below average in three or more subjects including Portuguese, in the last two quarters)
Which children are unable to keep up with the class, with tutoring or professional help having been recommended?

Of the 166 assessed children, 13 (7.8%) were appointed by the teacher and whose parents agreed with this appointment to participate in the study.

Finally, based on the questionnaire answered by the teacher, only 13 children with great difficulty in reading were selected for the study, which corresponded to 7.8% of the total population that was initially analyzed and included in the proposed criteria; there were 5 females and 8 males, with a mean age of 9 years and 9 months; 12 children were attending the 4th year of elementary school and one child was attending the 5th year of elementary school.

### Procedures

**Fig. 1:**
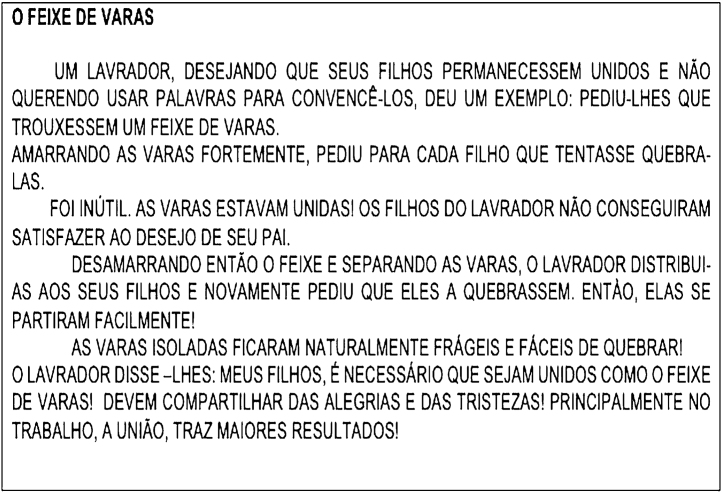
Text for reading assessment — “The Bundle of Sticks”. The children in GR1 did not read it because they were not literate yet.

The 13 children selected for the study were evaluated during a phoniatric consultation and then were classified into groups, according to reading skills and retelling of the text “The Bundle of Sticks” (Fig. 1). The reading was performed aloud by the child, during which fluency and decoding of graphic symbols were observed, while text comprehension was assessed through retelling. The examiner then read the text aloud and again assessed text comprehension through the child's retelling.

Fluency and decoding were considered very altered when the reading was performed with difficulty, showing problems in precision and speed, prolonged pauses and several changes and (or) omissions of phonemes. Fluency and decoding were considered little altered when the child showed some changes or omissions of phonemes that showed little impact on the speed and accuracy of reading. Fluency and decoding were considered adequate when the child showed good accuracy and reading speed and did not show phoneme changes and (or) omissions. This analysis was considered subjective and depended on the examiner's observation and judgment.

Based on the reading and retelling task, the children were grouped into 6 groups (GR):

GR1 — children who could not read the text. They were not literate yet. They did not retell the text after the examiner's reading.

GR2 — children who read the text but did not retell anything after their own reading or after the examiner’s reading.

GR3 — children who read the text and did not retell after their own reading; however, after the examiner’s reading, they retold the overall content of the text.

GR4 — children who read the text and were not able to retell any of the text after their own reading; however, after the reading was performed by the examiner, they were able to retell the entire text in details.

GR5 — children who read the text and retold the overall content of the text after their own reading.

GR 6 — children who read the text and retold the text in detail after their own reading.

Table 2 depicts the children who belong to each group. None of the children retold the text in details after reading it themselves, a necessary criterion to be included in GR6.

**Table 2:** Reading and retelling task: division of children into groups after the task (n = 13).

Groups	Children	Reading and retelling task^a^
GR1	2	Children who could not read the text. They were not literate. They did not retell after the examiner read the text.
GR2	2	Children who read the text but did not retell anything after their own reading or after it was read by the examiner.
GR3	2	Children who read the text and did not retell after their own reading; however, after it was read by the examiner, they retold the general content of the text.
GR4	4	Children who read the text and were not able to retell any of the text after their own reading; however, after the reading was performed by the examiner, they were able to retell the entire text in detail.
GR5	3	Children who read the text and retold the general content of the text after their own reading.
GR6	0	Children who read the text and retold the text in details after their own reading.^b^

a Read aloud by the child, where fluency and retelling was observed, and reading by the evaluator, where retelling was observed.

b GR6 — none of the children retold the text in details after their own reading.

Subsequently, the 13 selected children were evaluated by standardized and non-standardized tests used in the phoniatric assessment according to parameters published by Dualibi et al.^13^ The assessment of auditory and visual perceptual skills was analyzed through the application of 22 tests:

Figure naming test for children — this test is a short version of the 60 figure naming test by Seabra et al.^14^ The variable below the mean for age was used for the analysis.

Auditory discrimination test — assessed using a standardized technique for children aged 5–9 years, consisting of 30 pairs of syllables, with 10 equal pairs and 20 different pairs by Rodrigues.^15^ The altered variable was used for the analysis.

Syllable synthesis, phonemic synthesis, alliteration, syllable segmentation, phonemic segmentation, rhyme, syllabic manipulation, phonemic manipulation, syllabic transposition and phonemic transposition — standardized oral production phonological awareness skills tests for children aged 3–14 years by Seabra and Capovilla.^16^ The variable below average for age was used for the analysis.

Repetition of numbers in random order and repetition of numbers in reverse order — auditory working memory tests, carried out through the repetition of digits in random order and repetition of digits in reverse order, by Capellini et al.^17^ the altered variable was used for the analysis.

Pseudoword repetition — word and pseudoword repetition test by Seabra and Capovilla.^16^ The variable below average for age was used for the analysis.

Visual discrimination of letters and visual discrimination of words — evaluation of the visual discrimination of letters and words, using exercises of discrimination in letters and words proposed by Myklebust and Johnson.^18^ The altered variable was used for the analysis.

Visual memory — the standardized test from the book “Cognitive perceptual motor disfunction”, by Rubin et al.^19^ for children aged 6 years 2 months to 9 years and 7 months was performed. In children above this age, visual memory was considered impaired when it was below the highest age range standardized in the test. The child received 10 cards with one, three or four geometric figures of varying complexity. One card at a time was presented for 10 s; then, the examiner removed the form and the child had to draw the corresponding geometric shapes. The altered variable was used for the analysis.

Rapid figure naming — a test standardized by level of schooling by Capellini et al. was used.^17^ Naming tests require quick, successive and sequential evocation of symbols, as well as in reading, where there is rapid and successive decoding of symbols. In this test, for children attending the 4th and 5th years of elementary school, it is expected that the time spent to name the figures in sequence do not exceed 40″, being considered under attention when this time exceeds 50″. The figures used in this test are simple and common to children. It was considered adequate when the obtained result was within the expected level for the level of schooling; it was considered altered when the obtained result was “under attention” The altered variable was used for the analysis.

Visual synthesis with words — a non-standardized test proposed by the examiner was used. In this test, the child has to identify six words through scattered letters: plate, door, car, blue, plane, soap and pencil. The child was considered as having an adequate performance in the visual synthesis task when they were able to write most of the presented words (50% + 1); and altered when they could not analyze and write any of the words or less than 50% of the presented words. The altered variable was used for the analysis.

Figure copying — a test that assesses spatial organization in the graphic plane through the copying of geometric figures.^20^ The altered variable was used for the analysis.

### Statistical analysis

The data were initially descriptively analyzed due to the small sample size. Subsequently, the dendrogram statistical method was used, which is a quantitative method that organizes in a diagram the groupings comprising the variables and their levels of similarity,^21^ aiming to assess the correlation between altered or below-average results in auditory and visual perception tests. In the word repetition test, none of the children obtained below-average values; therefore, this variable was not considered in the remainder of the analysis and, therefore, 21 tests were considered in this analysis of groupings. Using this technique, the test results were grouped in such a way that those in the same group are more correlated with each other than with the tests in another group. The phi correlation coefficient was considered a measure of similarity and the grouping method adopted was the mean of distances.^22^ The phi correlation coefficient was also used to identify tests of which results are highly correlated.

Finally, the results obtained in the dendrogram with the grouping of tests were compared with the classification of the six groups according to reading and retelling skills. The discriminatory capacity of the groups was assessed individually, as the sample size does not allow the use of multivariate techniques.

## Results

### Descriptive analysis

Table 3 shows anamnesis data related to the subjects’ personal background, by groups, namely: speech delay; persistent speech disorders at six years of age, perinatal diseases, previous history of recurrent otitis, prematurity; previous speech therapy and previous psychological therapy. Other background data were not reported by the subjects during the phoniatric consultation and therefore are not included in the table.

**Table 3:** Descriptive summary of the children's personal background separated by groups.

Groups	Subjects	Speech delay	Other speech alterations	Perinatal conditions	Repeated otitis	Mild prematurity	Speech therapy	Psychotherapy
GR1	1	X					X	
	2							
GR2	3	X						
	4							
GR3	5	X		X		X	X	
	6			X		X		X
GR4	7						X	X
	8	X	X					X
	9	X	X					
GR5	10							X
	11							X
	12			X	X	X	X	X
	13							

There were no children with previous speech disorders in GR5 and children who had a history of persistent speech disorder at six years of age belonged to GR4, children with considerable decoding difficulty. It is also observed, in relation to personal history, that the two subjects who belonged to GR3 had perinatal diseases and mild prematurity.

Regarding therapy, it is shown in Table 3 that five children from different groups had never undergone any type of therapy, such as treatment for complaints of school difficulties, while eight children were undergoing or had undergone therapy, such as speech therapy and psychological therapy; among them was subject 12 from GR5, who also had a positive personal history of recurrent otitis.

As for family history, four children had a positive family history of language or learning problems; of these, two belonged to GR1, one to GR2 (subject 3) and another to GR5 (subject 10). Two children had a positive family history of psychological disorders, one from GR1 (subject 2) and the other from GR2 (subject 4).

### Fluency analysis and reading decoding

Table 4 represents the percentage of individuals in each group with alterations in fluency and decoding. This table shows that the three children from GR4 read with very altered fluency and decoding and this was the group with the worst performance among those who participated in the reading fluency and decoding assessment. The GR5 was the group with the best performance, and subjects 11 and 12, from this group, showed little altered fluency. It is also observed that all subjects in GR3 showed alterations in reading fluency. The two subjects from GR1 could not read.

**Table 4:** Percentages of individuals with little reading fluency and decoding alteration and with very altered fluency and decoding in each group defined by reading comprehension performance.

Group^a^	Little reading fluency and decoding alteration	Very altered fluency and decoding
GR 2	**50.0%**	**50.0%**
GR 3	**50%**	**50.0%**
GR 4	**0%**	**100.0%**
GR 5	50.0%	0.0%

a The two GR1 subjects did not read. All children in groups GR2, GR3 and GR4 showed alterations in fluency and reading decoding. Relevant data is shown in bold.

### Analysis of auditory and visual perception tests

**Fig. 2:**
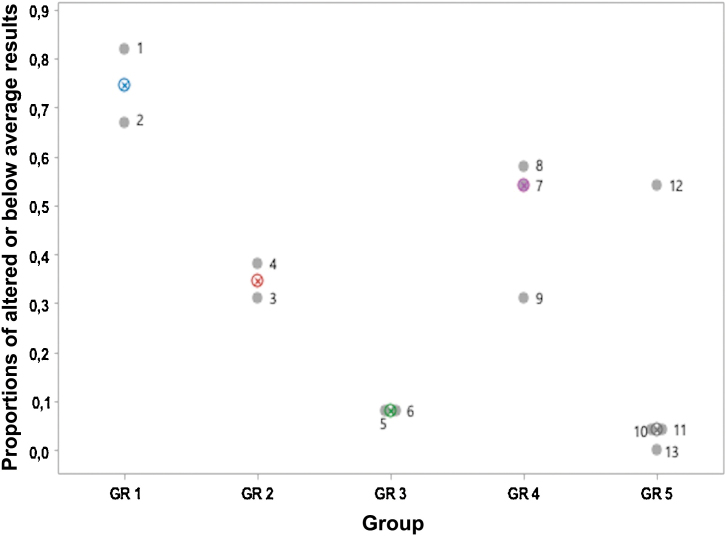
Individual and median proportions of altered or below average results in tests of auditory and visual perceptions and reading in the GR1 to GR5 groups. *The numbers in each point correspond to the children’s identification. **GR6 — none of the children retold the text in details after their own reading.

The two children in GR1 did not participate in the reading test, as they were not yet literate, and the individual totals were divided by the number of tests in which each child participated, thus obtaining, for each child, the proportion of altered or below-average results. Fig. 2 shows the proportion of the median of the altered test results in which each child participated; the proportion of altered results decreases from GR1 to GR3 and the only group with a median greater than GR4 is GR1 and the lowest median was observed in GR5. It is also observed that the medians in GR3 and GR5 are close. The points are identified by the child’s number in each group.

Fig. 2 shows that subject 12, despite belonging to GR5, that is, having better reading comprehension in comparison to GR4, has perceptual characteristics that are similar to those of GR4.

### Analysis of the discriminatory capacity of the tests

**Fig. 3:**
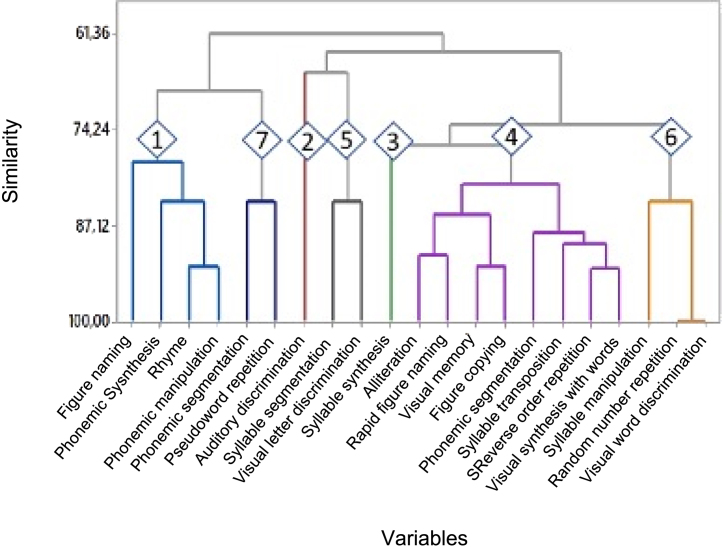
Dendrogram obtained from the analysis of the grouping of 21 variables related to tests of auditory and visual perceptions in which all subjects participated. *Grouping 1: figure naming, phonemic synthesis, rhyme, phonemic manipulation; grouping 2: auditory discrimination; grouping 3: syllable synthesis; grouping 4: alliteration, phonemic segmentation, syllable transposition, reverse order repetition, visual memory, visual synthesis with words, figure copying, rapid figure naming; grouping 5: syllable segmentation, visual letter discrimination; grouping 6: syllable manipulation, random number repetition, visual word discrimination; grouping 7: phonemic transposition, pseudoword repetition.

To identify the variables among the 21 tests, the results of those with similar behavior were grouped using the group analysis technique, which generated a dendrogram (Fig. 3) with seven groups of variables.

The discriminatory capacity of the tests was individually assessed considering the groupings. The percentages of individuals with an altered or below-average results in each of the variables in the different groupings are shown in Table 5, Table 6, Table 8, Table 9, Table 11, Table 12, Table 14. The phi correlation coefficient values ​​of variables in the same grouping are shown in Table 7, Table 10. The performance in the auditory discrimination and syllabic synthesis tests showed a poor correlation with the other tests. These results suggest that:

**Table 5:** Grouping 1: percentage of individuals with altered or below-average results in the figure naming, phonemic synthesis, rhyme, and phonemic manipulation tests in groups GR1 to GR5.

Group	Figure naming	Phonemic synthesis	Rhyme	Phonemic manipulation
GR 1	**50.0%**	**100.0%**	**100.0%**	**100.0%**
GR 2	**50.0%**	**100.0%**	**50.0%**	**50.0%**
GR 3	0.0%	0.0%	0.0%	0.0%
GR 4	**33.3%**	**66.7%**	**33.3%**	**66.7%**
GR 5	0.0%	50.0%	25.0%	25.0%
Total	23.1%	61.5%	38.5%	46.2%

^a^The tests in this grouping discriminate the groups GR1, GR2 and GR4 from the others. Relevant data are shown in bold.

**Table 6:** Grouping 2: percentage of individuals with altered results in the auditory discrimination test in groups GR1 to GR5.

Group	Auditory discrimination
GR 1	50.0%
GR 2	50.0%
GR 3	50.0%
**GR 4**	**100.0%**
GR 5	25.0%
Total	53.8%

^a^The auditory discrimination test discriminates the GR4 from the others. The relevant data are shown in bold.

**Table 7:** Grouping 1: phi correlation coefficients of the figure naming, phonemic synthesis, rhyme and phonemic manipulation tests.

	Figure naming	Phonemic synthesis	Rhyme	Phonemic manipulation
Figure naming	1.00	0.43	0.69	0.59
Phonemic synthesis	0.43	1.00	0.63	**0.73**
Rhyme	0.69	0.63	1.00	**0.85**
Phonemic manipulation	0.59	**0.73**	**0.85**	1.00

^a^There is a strong correlation between the phonemic synthesis and phonemic manipulation tests, and between phonemic manipulation and rhyme. The figure naming test shows a moderate correlation with phonemic synthesis and phonemic manipulation. Relevant data are shown in bold.

**Table 8:** Grouping 3: percentage of individuals with altered results in the syllabic synthesis test in groups GR1 to GR5.

Group	Syllabic synthesis
**GR 1**	**50.00%**
GR 2	0.00%
GR 3	0.00%
**GR 4**	**33.30%**
GR 5	0.00%
Total	15.40%

^a^The syllabic synthesis test discriminates GR1 and GR4 from the others. Relevant data are shown in bold.

**Table 9:** Grouping 4: percentage of individuals with altered or below-average results in the tests of alliteration, phonemic segmentation, syllable transposition, reverse-order repetition, visual memory, visual synthesis with words, figure copying, and rapid figure naming in groups GR1 to GR5.

Group	Alliteration	Phonemic segmentation	Syllable transposition	Reverse-order repetition	Visual memory	Visual synthesis with words	Figure copying	Rapid figure naming
**GR 1**	**100.0%**	**100.0%**	**100.0%**	**100.0%**	**100.0%**	**100.0%**	**100.0%**	**100.0%**
**GR 2**	**50.0%**	**50.0%**	**0.0%**	**50.0%**	**50.0%**	**100.0%**	**50.0%**	**0.0%**
GR 3	0.0%	0.0%	0.0%	0.0%	0.0%	0.0%	0.0%	0.0%
**GR 4**	**33.3%**	**100.0%**	**66.7%**	**66.7%**	**33.3%**	**66.7%**	**33.3%**	**33.3%**
GR 5	0.0%	25.0%	25.0%	25.0%	50.0%	25.0%	25.0%	0.0%
Total	30.8%	53.8%	38.5%	46.2%	46.2%	53.8%	38.5%	23.1%

^a^The tests of alliteration, phonemic segmentation, syllable transposition, reverse order repetition, visual memory, visual synthesis with words, figure copying, and rapid figure naming discriminated GR1, GR2 and GR4. Relevant data are shown in bold.

**Table 10:** Grouping 4: phi correlation coefficients of the tests of alliteration, phonemic segmentation, syllable transposition, reverse order repetition, visual memory, visual synthesis with words, figure copying, and rapid figure naming. The dark gray lines refer to data with high correlation with the tests.

	Alliteration	Phonemic segmentation	Syllable transposition	Reverse order repetition	Visual memory	Visual synthesis with words	Figure copying	Rapid figure naming
Alliteration	1.00	0.62	0.50	**0.72**	**0.72**	0.62	**0.84**	**0.82**
Phonemic segmentation	0.62	1.00	**0.73**	**0.86**	0.55	0.69	**0.73**	0.51
Syllable transposition	0.50	**0.73**	1.00	**0.85**	0.54	**0.73**	0.68	0.69
Reverse Order repetition	**0.72**	**0.86**	**0.85**	1.00	0.69	**0.86**	**0.85**	0.59
Visual memory	**0.72**	0.55	0.54	0.69	1.00	0.55	**0.85**	0.59
Visual synthesis with words	0.62	0.69	**0.73**	**0.86**	0.55	1.00	0.73	0.51
Figure copying	**0.84**	**0.73**	0.68	**0.85**	**0.85**	**0.73**	1.00	0.69
Rapid figure naming	**0.82**	0.51	0.69	0.59	0.59	0.51	0.69	1.00

**Table 11:** Grouping 5: percentage of individuals with altered or below-average results in the syllable segmentation and visual letter discrimination tests in groups GR1 to GR5.

Group	Syllable segmentation	Visual letter discrimination
GR 1	50.0%	0.0%
GR 2	0.0%	0.0%
GR 3	0.0%	0.0%
GR 4	0.0%	0.0%
GR 5	**25.0%**	**25.0%**
Total	15.4%	7.7%

The syllable segmentation and visual letter discrimination tests did not discriminate between the groups. *Relevant data are shown in bold.

**Table 12:** Grouping 6: percentage of individuals with altered or below-average results in the syllable manipulation, random order repetition and visual word discrimination tests in groups GR1 to GR5.

Group	Syllable manipulation	Random order repetition	Visual word discrimination
GR 1	**50.0%**	**100.0%**	**100.0%**
GR 2	0.0%	0.0%	0.0%
GR 3	0.0%	0.0%	0.0%
GR 4	0.0%	0.0%	0.0%
GR 5	0.0%	0.0%	0.0%
Total	7.7%	15.4%	15.4%

^a^The syllable manipulation, random order repetition and visual word discrimination tests discriminate GR1 from the other groups. Relevant data are shown in bold.

In grouping 1: the figure naming, phonemic synthesis, rhyme and phonemic manipulation tests discriminated the groups GR1, GR2 and GR4 from the others (Table 5). There is a strong correlation between the phonemic synthesis and phonemic manipulation tests, and between phonemic manipulation and rhyme (Table 7); they were the ones with the highest phi correlation coefficient value, with both being significant. The figure naming test showed a moderate correlation with phonemic synthesis and phonemic manipulation (Table 7). Most of the children from GR1, GR2 and GR4 showed alterations in phonemic synthesis, phonemic manipulation, rhyme and figure naming. These children represent those who had difficulty retelling the text after their own reading. They are the ones that showed the worst performance in reading, either due to decoding difficulty, as those in GR4, comprehension difficulty, as those in GR2 or due to both difficulties, as those in GR1. Although the existing difficulties among the children are diverse, this set of tests showed sensitivity to identify children with the greatest difficulties in reading.

In grouping 2: the auditory discrimination test discriminated the GR4 from the others (Table 6). The auditory discrimination, although it did not show a significant correlation with the other tests, showed good sensitivity to identify children in GR4, being altered in all of them.

In grouping 3: the syllabic synthesis test discriminated GR1 and GR4 from the others (Table 8 ). In this case, an alteration was observed only in the children from GR1 and GR4, the groups to which the children with greater difficulties in decoding belong.

In grouping 4: the alliteration, phonemic segmentation, syllable transposition, repetition of numbers in reverse order, visual memory, visual synthesis with words, figure copying, and rapid figure naming tests discriminated GR1, GR2 and GR4 (Table 9). The high correlations between the repetition of numbers in reverse order (working memory) and figure copying with most variables in this grouping stand out (Table 10).

In grouping 5: the results shown in Table 11 suggest that the syllable segmentation and visual letter discrimination tests did not adequately discriminate the groups.

In grouping 6: the results shown in Table 12 suggest that the syllable manipulation, random number repetition and visual word discrimination tests discriminated GR1 from the others. The phi correlation coefficient value between the random number repetition and the visual word discrimination tests is equal to 1 (Table 13), that is, the individuals in the sample showed the same response in both tests.

**Table 13:** Grouping 6: phi correlation coefficients of the syllable manipulation, random order repetition, and visual word discrimination tests.

	Syllable manipulation	Random order repetition	Visual word discrimination
Syllable manipulation	1.00	0.68	0.68
Random order repetition	0.68	**1.00**	**1.00**
Visual word discrimination	0.68	**1.00**	**1.00**

^a^The value of the phi correlation coefficient between the random order repetition and the visual word discrimination tests is equal to 1, that is, the individuals in the sample showed the same response in both tests. *Relevant data are shown in bold.

In grouping 7: The results shown in Table 14 suggest that the phonemic transposition and pseudoword repetition tests did not discriminate between the groups.

**Table 14:** Grouping 7: percentage of individuals with below-average results in the phonemic transposition and pseudoword repetition tests in groups GR1 to GR5.

Group	Phonemic transposition	Pseudoword repetition
GR 1	50.0%	0.0%
GR 2	0.0%	0.0%
GR 3	0.0%	0.0%
GR 4	**33.3%**	**33.3%**
GR 5	0.0%	0.0%
Total	15.4%	7.7%

^a^The phonemic transposition and pseudoword repetition tests did not discriminate between the groups. Relevant data are shown in bold.

## Discussion

In this study, the children attended a school of excellence standards, contributing to the validity of the schooling of the studied group. School adequacy was an important factor, because in the discussion of a diagnostic hypothesis for children with learning disabilities, the conditions of the school environment should be discarded as a cause.

Thirteen children with great reading difficulty were assessed, corresponding to 7.8% of the population initially included in the study; this percentage is in accordance with the prevalence mentioned in the DSM-5 for the specific learning disorder, which ranges from 5% to 15% for school-age children, in different languages ​​and cultures.^4^

Reading comprehension is a complex process, supported not only by the identification of written words and vocabulary but also by language systems, such as syntax and overall knowledge. Thus, reading comprehension impairments can develop as a result of a deficit in cognitive and/or linguistic neural areas.^7^, ^8^ Therefore, we consider that the retelling task, not only after reading by themselves, but also after the examiner’s reading, is of great importance, as it infers a broader language comprehension beyond the phonological domain. This task allowed the distinction of groups based on reading comprehension and contributed to the thinking of individual therapeutic approaches.

The children from GR3 and GR4 improved their comprehension after the examiner's reading, suggesting that reading fluency is the cause of the comprehension difficulty. On the other hand, children from GR2, who did not improve their comprehension after reading was performed by the examiner, seem to have difficulties related to higher cognitive processes, which impair their understanding. Oakhill et al. showed that the ability to understand the meaning of reading depends, in addition to decoding skill, on the capacity of metacognitive monitoring, textual integration, knowledge of text structure and working memory, and the authors recognize that both skills are important for reading comprehension.^8^

Regarding the analysis of the alterations found in the tests performed to assess auditory and visual perceptions, a worse performance was observed, in general, in groups GR1, GR2 and GR4. Children from GR3 who failed to retell the text after their own reading and children from GR5 who were able to retell the text, in general, after reading themselves, showed few alterations in the auditory and visual perception tests. This result leads us to suppose that their difficulties may be due to environmental factors or daily life habits, such as lack of training in reading.

Subject 12, specifically, identified by the teacher as having school difficulties, underwent speech and psychopedagogical therapy and stands out for having shown a good performance in reading and a poor performance in the auditory and visual perception tests. We believe that the therapeutic processes may have contributed to their reading performance, although the difficulties related to other skills still compromise their school performance.

Snowling & Melby-lervåg,^3^ stated that learning how to read also depends on training and the more a child reads, the more their vocabulary and spelling are improved; therefore, the excess of electronic media and the lack of encouragement to read can be considered as the cause of school difficulties in children with neurological conditions that are adequate for learning.

The syllabic synthesis test discriminated the groups GR1 and GR4 from the others (Table 8) and the auditory discrimination was altered in all the children from GR4 (Table 6), suggesting that the auditory discrimination may be an indicator of an alteration more related to the phonological route in children with important decoding difficulties.

Some tests were able to differentiate the groups GR1, GR2 and GR4 from the others with more sensitivity, namely: figure naming, phonemic synthesis, rhyme, phonemic manipulation, alliteration, phonemic segmentation, repetition of numbers in reverse order, visual synthesis with words and figure copying. One can consider that children belonging to these three groups and, therefore, who show more alterations in these tests, may lead the clinician to a diagnostic hypothesis of learning disorder.^4^ However, we emphasize that the differentiation between the types of difficulties, as it occurs between GR1, GR2 and GR4 is essential, as it implies different therapies.

A high correlation is observed between alliteration, phonemic segmentation, syllabic transposition, visual word synthesis and figure copying with the repetition of numbers in reverse order (working memory) test, a task that demands working memory/attention (Table 10). There was a strong correlation between this test and figure copying, with most of the variables in grouping 4 (Table 10). Overall, the findings in these groups suggest prioritizing the performance of the repetition of numbers in reverse order and figure copying tests.

The syllabic synthesis test (Table 8) discriminated, in our study, children from GR1 and GR4 (children with greater decoding problems) and the following tests: figure naming, phonemic synthesis, rhyme and phonemic manipulation discriminated between GR1, GR2 and GR4. Mourão Junior and Melo observed that children with specific learning disabilities in reading and writing have deficits in the phonological loop of working memory; these authors suggest that learning deficits may actually be executive deficits, therefore related to attention, with working memory, or with the inhibitory control.^9^ Therefore, they assume that children who do not learn may not be able to use what they learned, and they consider that perhaps therein lies the real origin of the problem, and a therapeutic approach may emerge from that situation.

The strong relationship between most phonological awareness tests and working memory may raise some questions: would the alterations in executive functions and, more specifically in working memory, be responsible for the inadequate performance in the phonological awareness tests shown by these children? Could we then attribute learning disorders to deficits in executive functions, as suggested by some abovementioned authors? And, in these cases, could children with more global deficits in executive functions have greater difficulties in reading comprehension, as they are unable to efficiently maintain selective, sustained attention, inhibitory control and working memory to sustain all of their perceptual learning, which is necessary for learning?

The coherence of the results and the relationship between the proposed tasks suggest that reading tasks with retelling, both after the patient’s reading and after the examiner's reading, when analyzed together, can guide the assessment as a whole. Based on the analysis of the similarity between the performances in the 21 perceptual tests applied to the groups classified according to their reading difficulty, it was possible to suggest the ones that can be considered more relevant in the process of diagnosing children with complaints of learning difficulties in reading.

Overall, our findings suggest that the steps: (1) reading assessment with retelling tasks and fluency observation; (2) test of repetition of numbers in reverse order; (3) figure copying test; (4) figure naming test; (5) assessment of phonological awareness (syllabic synthesis, phonemic synthesis, rhyme and phonemic manipulation) contribute to aspects of the diagnosis and multidisciplinary interventions.

This study had some limitations regarding population and sample size; the method used to identify children with reading difficulties was based on interviews with teachers and the inclusion and exclusion criteria may have contributed to sample size limitation and the creation of small groups, according to the performance in the reading task. Moreover, the number of standardized and non-standardized tests applied may have interfered with the interpretation of the statistical correlation analysis with the groups, with the quantitative method of cluster analysis by similarity being considered more appropriate. And finally, the population of assessed children had different clinical conditions that may have directly influenced the result. Therefore, there was no intention to establish an assessment protocol, but to appraise the performance diversity and, consequently, the conduct in children with similar complaints.

Considering that phoniatrics is essentially clinical, the theoretical training, experience and self-knowledge of the specialist in phoniatrics, together with the appreciation of the uniqueness of each new case, should promote new studies that expand and support this clinic. Therefore, it is expected that this study may contribute to a greater understanding of school-age children’s complaints in clinical phoniatrics and assist future research on learning disorders.

## Conclusion

In the context of the phoniatric consultation, this study evaluated the task of reading and retelling by children with relevant reading difficulties who could potentially seek a consultation with a specialist in phoniatrics, grouping them according to their performance and correlating these groups with auditory and visual perceptual tests, suggesting that the naming of figures, repetition of numbers in reverse order, copying of figures, syllable synthesis, phonemic synthesis, rhyme and phonemic manipulation tests can contribute to aspects of the diagnosis of learning difficulties.

## Funding

Vanessa Magosso Franchi received financial support from the 10.13039/501100002322Coordination for the Improvement of Higher Education Personnel — Brazil (CAPES), process number 88887.151901/2017-00.

## Conflicts of interest

The authors declare no conflicts of interest.
